# Draft Genome Sequence of Aquincola tertiaricarbonis MIMtkpLc11, an Aerobic Anoxygenic Phototrophic Bacterial Strain Isolated from Biological Soil Crusts

**DOI:** 10.1128/MRA.01085-18

**Published:** 2018-09-27

**Authors:** Kai Tang, Bo Yuan, Yonghui Zeng, Lijuan Jia, Fuying Feng

**Affiliations:** a Institute for Applied and Environmental Microbiology, Life Sciences College, Inner Mongolia Agriculture University, Huhhot, China; b Life Sciences and Technology, College of Inner Mongolia Normal University, Huhhot, China; c Aarhus Institute of Advanced Studies, Aarhus University, Aarhus, Denmark; d Department of Environmental Science, Aarhus University, Roskilde, Denmark; Loyola University Chicago

## Abstract

Aquincola tertiaricarbonis strain MIMtkpLc11 was isolated from biological soil crusts in Inner Mongolia, China. The strain contains photosynthesis gene clusters. Here, we report the draft genome sequence of strain MIMtkpLc11, which comprises 98 contigs (*N*_50_, 233,472 bp) and 5,573 protein-coding sequences.

## Abstract (precis)

Aquincola tertiaricarbonis strain MIMtkpLc11 was isolated from biological soil crusts in Inner Mongolia, China. The strain contains photosynthesis gene clusters.

## ANNOUNCEMENT

Biological soil crusts (BSCs) are widely distributed in dry lands (1). They show the ability to preserve soil fertility and reduce erosion (2), and they play a significant role in global carbon cycles (3). Aerobic anoxygenic phototrophic bacteria belong to the classes Alphaproteobacteria, Betaproteobacteria, and Gammaproteobacteria and contribute significantly to the recycling of organic matter (4). Aquincola is a genus in the class Betaproteobacteria, and its members have been reported to degrade tertiary butyl moieties (5) and possess the photosynthesis gene cluster (comprising *bchFNBHL*, *ppaA*, *ppsR*, and *bchG*) (6). Presently, the genus Aquincola has only one type species (5), and genomic data on its members are still lacking.

Strain Aquincola tertiaricarbonis MIMtkpLc11 was isolated from BSCs in Liangcheng, Inner Mongolia, China. This white-transparent strain was isolated by plating diluted BSCs onto R2A agars at 28°C. The 16S rRNA gene was amplified with the universal bacterial primers 27F and 1492R (7) and assembled using SeqMan version 7 software (8). Similarities between the 16S rRNA gene sequence of A. tertiaricarbonis strain MIMtkpLc11 and the closest relative species were calculated with EZBioCloud (9; see also http://www.ezbiocloud.net), and the results showed the highest similarity to be 98.36% with the type strain A. tertiaricarbonis L10 (GenBank accession number NR_043913).

Genomic DNA was extracted using a genomic DNA purification kit (Tiangen Biotech Co., Beijing, China) and fragmented to sizes of 300 to 500 bp using the M220 ultrasonicator (Covaris, USA). Paired-end libraries (300-bp length) were prepared using a TruSeq DNA sample prep kit (Illumina, Inc., USA) and then sequenced using the Illumina HiSeq 2000 system. Prior to genome assembly, the raw reads were trimmed using Trimmomatic version 3.02 (10). The clean reads were assembled and corrected with SOAPdenovo version 2.04 (11) and GapCloser version 1.12 (12) under the default settings. The open reading frames (ORFs) of the assembled genome were predicted with Glimmer version 3.02 (13). All predicted ORFs were then searched with BLAST version 2.2.30+ (14) against all proteins from complete microbial genomes in the NCBI Prokaryotic Genome Annotation Pipeline (PGAP) version 3.1 (15).

The raw sequence data comprise a total of 8,901,302 reads and 1,121,564,052 bases, which provide a high sampling coverage of the genome (169-fold coverage). The draft genome comprises 98 contigs with an *N*_50_ value of 233,472 bp and an *N*_90_ contig length of 54,404 bp. The total length is 6.32 Mb, containing 5,573 protein-coding sequences (CDSs), 3 rRNAs (including 1 16S rRNA, 1 23S rRNA, and 1 5S rRNA), and 43 tRNAs (19 types). The G+C content was 70.18%. The photosynthesis gene cluster contains *pufABCLM*, *bchCFMOXYZ*, *crtBCDFIWXZ*, *pucC*, *puhA*, and *acsF*. The *pufM* protein sequences were compared against the NCBI protein database and showed 85% and 86% similarities to those of Roseateles terrae (GenBank accession number OWQ86667) and Pelomonas sp. HMWF004 (GenBank accession number PTT84989), respectively.

### Data availability.

The genome sequence of A. tertiaricarbonis strain MIMtkpLc11 has been deposited in DDBJ/EMBL/GenBank under the accession number JZUE00000000. The version described in this paper is the first version, JZUE01000000. The raw sequences were deposited in the Sequence Read Archive (SRA) under the accession number SRP159127.
